# HIV testing among incarcerated people with a history of HIV-related high-risk behaviours in Iran: Findings from three consecutive national bio-behavioural surveys

**DOI:** 10.1186/s12879-022-07897-z

**Published:** 2022-12-05

**Authors:** Fatemeh Tavakoli, Najmeh Parhizgari, Mostafa Shokoohi, Mehrdad Khezri, Ali Akbar Haghdoost, Iman Ghasemzadeh, Ivana Bozicevic, Armita Shahesmaeili, Naser Nasiri, Ahmad Danesh, Mohammad Karamouzian, Hamid Sharifi

**Affiliations:** 1 HIV/STI Surveillance Research Center, and WHO Collaborating Center for HIV Surveillance, Institute for Futures Studies in Health, Kerman University of Medical Sciences, Kerman, Iran; 2 Medical Virology Department, School of Public Health, Tehran University of Medical Sciences, Tehran, Iran; 3 Dalla Lana School of Public Health, University of Toronto, Toronto, ON Canada; 4 WHO Collaborating Centre for HIV Strategic Information, School of Medicine, Zagreb, Croatia; 5 Golestan Research Center of Psychiatry, Golestan University of Medical Sciences, Gorgan, Iran; 6 Centre on Drug Policy Evaluation, St. Michael’s Hospital, Toronto, ON Canada; 7 Brown School of Public Health, Brown University, Providence, RI USA

**Keywords:** HIV testing, Prison, Sexual behaviour, Injection drug use, Iran

## Abstract

**Background:**

Incarcerated people are at a disproportionate risk of contracting HIV. We estimated the prevalence and correlates of HIV testing among incarcerated people with a history of HIV-related high-risk behaviours in Iran.

**Methods:**

Data for this analysis were obtained from three consecutive nationwide bio-behavioural surveillance surveys of a random sample of incarcerated people in 2009 (n = 5953), 2013 (n = 5490), and 2017 (n = 5785). History of testing for HIV in the last 12 months was the primary outcome variable. HIV testing was examined among those with a history of HIV-related high-risk behaviours (i.e., having multiple sex partnerships, injection drug use practices, or a history of having a tattoo). The outcome variable was divided into three categories: Never tested for HIV, ever tested for HIV inside the prison in the last 12 months, and ever tested for HIV outside the prison in the last 12 months. We used multivariable multinomial logistic regression models to examine factors associated with HIV testing.

**Results:**

Overall, 8,553 participants with a history of HIV-related high-risk behaviors with valid responses to the HIV testing question were included in the analysis. Although HIV testing inside prison has increased (23% [2009], 21.5% [2013], and 50.3% [2017]: P-value < 0.001), the prevalence of HIV testing outside prison has decreased (7.7% [2009], 7.5% [2013], 4.1% [2017]: P-value < 0.001) over time. Our multivariable multinomial regression model showed older age (Relative-risk ratio [RRR]: 1.24, 95% Confidence Intervals [CI]: 1.05, 1.47), history of the previous incarceration (RRR: 1.46, 95% CI: 1.24, 1.71), currently receiving methadone maintenance therapy inside prison (RRR: 2.09, 95% CI: 1.81, 2.43), having access to condoms inside prison (RRR: 1.42, 95% CI: 1.20, 1.68) and sufficient HIV knowledge (RRR: 1.74, 95% CI: 1.47, 2.05) were significantly associated with an increased probability of having an HIV test in the last 12 months inside prison.

**Conclusion:**

HIV testing among high-risk Iranian prisoners has increased from 2009 to 2017. However, HIV testing remains considerably low, and half of the incarcerated people with a history of HIV-related high-risk behaviours had never tested for HIV inside prison. Evidence-based programs are needed to optimize HIV testing inside and outside prisons and identify those at greater risk of HIV.

**Supplementary Information:**

The online version contains supplementary material available at 10.1186/s12879-022-07897-z.

## Background

Incarcerated people are at a higher risk of contracting or transmitting HIV than the general population, primarily due to their engagement in HIV-related high-risk behaviours (e.g., injection drug use, tattooing [1], and unprotected sexual practices) as well as the populated nature of prison settings [2–4]. Incarcerated people could also be a bridging population and help transmit HIV to other subpopulations, such as their partners [5–8].

In Iran, where 97% of incarcerated people are men [9], about half of the incarcerated people face drug-related charges, and up to 70% use illicit drugs [4, 10–13]. In particular, incarcerated people who inject drugs (PWID) play an essential role in spreading HIV inside the prisons through sharing needles and syringe practices [14]. The first HIV outbreak inside prisons in Iran occurred in 1998 [15], documenting an all-time high HIV prevalence of 4.5%. After the outbreak, HIV prevalence was reported to be as high as 13.2% across certain prison settings [15]. As a response to the growing HIV epidemic among incarcerated people in Iran, harm reduction programs and triangular clinics were initiated in 2002 and scaled up across several major prisons in the country [16, 17]; interventions that helped curb the epidemic and reduce the HIV prevalence among incarcerated people to 2.1% in 2009 [12, 18]. Harm reduction services inside prisons in Iran include methadone maintenance therapy (MMT), free condoms for conjugal visits, and HIV testing and counseling [17, 19, 20]. Incarcerated people who report a history of HIV-related high-risk behaviours are provided with a voluntary HIV test upon entrance to the prison. Once inside prisons, HIV tests are available through both voluntary provider-initiated and client-initiated testing inside prison health clinics. HIV testing and treatment services outside prisons are free and voluntary for all [21, 22].

Despite the importance of assessing HIV testing rates among incarcerated people as a key population at risk of HIV, most previous studies in Iran have highlighted the unmet need for HIV testing among female sex workers (FSW) and PWID [23–25]. Harm reduction services, including voluntary counseling and HIV testing, were rapidly scaled up across prison settings in all provinces in Iran [26]. However, studies on HIV testing among incarcerated people in Iran remain limited, and data on the actual coverage of these services are rare. This study utilizes data from three repeated bio-behavioural surveillance surveys (BBSS) in 2009, 2013, and 2017 to assess HIV testing among a random sample of incarcerated people with a history of HIV-related high-risk behaviours. We also examined factors associated with HIV testing among incarcerated people in Iran.

## Methods

### Study setting and data collection

Three national BBSS were conducted among Iranian incarcerated people in 2009, 2013, and 2017. The design, sampling, and data collection approaches were harmonized across the three surveys and have been described elsewhere [12]. A multi-stage cluster sampling approach was designed to reduce the design effect and increase the precision of the study. Based on the median number of incarcerated people in each prison facility, the prisons in the country were first divided into two large and small categories to address the intra-class correlation due to the size of the prison. The number of samples needed in large and small prisons was determined according to the population of each category. In brief, the prison settings were categorized into two strata of large (n = 16) and small (n = 17) prisons based on the median number of incarcerated people. In 2009, 533 participants from small and 5380 participants from large prisons were recruited. The respective sample sizes for these two strata were 549 and 4881 participants in 2013 and 1120 and 4665 participants in 2017. Data in three surveys were collected through a multi-stage random sampling approach. Systematic random sampling was used to recruit eligible incarcerated people.

The eligibility criteria across all surveys were being incarcerated for at least one week, having not participated in a similar study in the previous two months, and providing verbal informed consent [27]. Gender-matched trained interviewers conducted face-to-face interviews in a private room inside the prisons. Although the sections in the questionnaire were similar across the three surveys in 2009, 2013, and 2017, slight modifications were applied to the questionnaire in 2013 and 2017 based on the feedback received from regional experts and the Ministry of Health.

### Outcome variable

As routine HIV testing is recommended for people at a high risk of HIV, we restricted the analysis to those with a history of HIV-related high-risk behaviours, including a history of injection drug use, multiple-sex partnerships (two or more partners at the same time), and having had a tattoo (ever). The primary outcome variable in this study was *“having completed an HIV test in the last 12 months”*. The primary outcome had three options (never tested vs. tested inside a prison in the last 12 months vs. tested outside a prison in the last 12 months). Incarcerated people were questioned: *“Have you ever tested for HIV?”* If no, they were coded as “never tested.” If yes, they were asked, *“When did you last test for HIV?”* and *“Where did you last test for HIV?”.* Participants who had a test during the last 12 months and inside the prison were considered to be “tested inside a prison in the last 12 months”. Participants who had a test during the last 12 months and outside the prison were considered to “test outside a prison in the last 12 months”.

### Covariates

Socio-demographic factors of interest were sex (male vs. female), age at interview (≤ 29 vs. >29) [28, 29], current marital status (single [i.e., never married] vs. married vs. other [i.e., divorced, widowed or temporary marriage/sigheh]), educational level (illiterate or primary school vs. secondary school or high school vs. college education).

Data were also recorded on drug use and sexual risk behaviours, history of previous incarcerations (yes vs. no), lifetime history of alcohol consumption (yes vs. no), lifetime history of illicit drug use (yes vs. no), current receipt of MMT (yes vs. no), having access to sterile syringe inside prison (yes vs. no), age at first sex (< 18 vs. ≥18 years), and having access to condom inside prison (yes vs. no). We also measured HIV knowledge (sufficient vs. insufficient [eight questions were asked about HIV transmission; >4 correct responses were coded as sufficient and otherwise as insufficient]) and self-perceived risk of HIV (yes vs. no).

### Statistical analysis

Descriptive statistics, including frequencies, percentages, and 95% confidence intervals (95% CI), were reported for HIV testing inside and outside of prison in the last 12 months. People living with HIV who knew their HIV status were excluded from the analysis. Bivariable and multivariable multinomial logistic regression models were built to compare the probability of having an HIV test in the last 12 months among different subgroups of incarcerated people after merging the data from three rounds of surveys and adjusting for the year of data collection. Variables with a p-value less than < 0.2 in the bivariable multinomial logistic regression model were entered into the multivariable multinomial logistic regression model. The final model was chosen through the backward elimination method using Wald statistics. Survey analysis using the Svy package in Stata /SE 14.0 (Stata Corp LP; College Station, Texas, USA) was used to analyze the data. Analyses were adjusted for prison’s sample sizes by applying appropriate sampling weights. A sensitivity analysis was conducted to assess correlates of HIV testing across different surveys in 2009, 2013, and 2017 through separate regression analyses (Additional file 1).

## Results

### Demographic characteristics and the prevalence of HIV testing

Of the 5953 participants in the 2009 survey, 3364 (56.5%) participants had a history of HIV-related high-risk behaviours (4.0% with a history of injection drug use, 46.3% having a tattoo, and 49.7% having multiple sex partners). Additionally, of the 5490 participants in 2013, 3,449 (62.8%; 5.6% with a history of injection drug use, 53.5% having a tattoo, 40.9% having multiple sex partnership), and out of 5785 participants in 2017, 3,353 (58.0%; 12.9% with a history of injection drug use, 48.9% having a tattoo, and 38.2% having multiple sex partnership) had a history of HIV-related high-risk behaviours and were included in this analysis. Among the 2009 survey participants, 97.6% (n = 2811) were male, 52.1% (n = 1500) were > 29, 45.7% (n = 1309) were single, and 55.5% (n = 1598) had secondary or high school education. In the 2013 survey, 98.8% (n = 2770) were male, 69.7% (n = 1945) were in > 29 years, 46.2% (n = 1294) were married, 51.9% (n = 1451) were secondary or high school education. In the 2017 survey, 96.1% (n = 2756) were male, 72.9% (n = 2086) were in > 29 years, 41.3% (n = 1185) were single, 61.8% (n = 1770) had secondary or high school education (Table 1).

**Table 1:** HIV testing inside and outside a prison in the last 12 months among incarcerated people

Variables	HIV testing inside and outside prison in the last 12 months					
	2009 (N = 2880)		2013 (N = 2804)		2017 (N = 2869)	
	Inside prison	Outside prison	Inside prison	Outside prison	Inside prison	Outside prison
	n (%)	n (%)	n (%)	n (%)	n (%)	n (%)
Sex						
Male	647 (23.0)	218 (7.7)	593 (21.4)	208 (7.5)	1363 (49.5)	117 (4.2)
Female	14 (20.3)	5 (7.2)	10 (29.4)	3 (8.8)	79 (69.9)	2 (1.8)
Age at interview						
≤29	249 (18.1)	91 (6.6)	134 (15.8)	65 (7.7)	329 (42.5)	38 (4.9)
>29	412 (27.5)	132 (8.8)	469 (24.1)	144 (7.4)	1110 (53.2)	80 (3.8)
Current marital status						
Single	284 (21.7)	78 (5.9)	265 (23.5)	78 (6.9)	587 (49.5)	61 (5.1)
Married	279 (22.1)	108 (8.5)	226 (17.5)	114 (8.8)	518 (46.7)	36 (3.2)
Divorced, widowed, or sigheh ^1^	94 (31.7)	33 (11.1)	112 (29.4)	19 (4.9)	337 (58.6)	22 (3.8)
Educational level						
Illiterate or primary school	282 (23.2)	79 (6.5)	287 (22.8)	67 (5.3)	482 (50.5)	27 (2.8)
Secondary or high school	358 (22.4)	136 (8.5)	300 (20.7)	136 (9.4)	894 (50.5)	85 (4.8)
College education	21 (30.4)	8 (11.6)	13 (15.1)	8 (9.3)	65 (47.4)	6 (4.4)
History of previous incarceration						
No	253 (22.5)	89 (7.9)	131 (14.3)	78 (8.5)	381 (42.3)	28 (3.1)
Yes	340 (24.0)	120 (8.4)	472 (25.0)	133 (7.0)	1061 (53.9)	91 (4.6)
Lifetime history of alcohol consumption						
No	NA ^*^	NA	85 (16.2)	47 (8.9)	260 (42.5)	17 (2.9)
Yes	NA	NA	517 (22.7)	164 (7.2)	1164 (52.0)	99 (4.4)
Lifetime history of drug use						
No	47 (11.0)	19 (4.5)	35 (9.1)	39 (10.2)	152 (38.9)	21 (5.4)
Yes	614 (25.0)	204 (8.3)	568 (23.5)	172 (7.1)	1290 (52.1)	98 (4.0)
Currently receiving MMT						
No	127 (20.3)	62 (9.9)	277 (17.9)	112 (7.2)	235 (52.5)	20 (4.5)
Yes	342 (38.0)	80 (8.8)	290 (33.2)	59 (6.7)	676 (56.8)	46 (3.8)
Had access to sterile syringe in prison						
No	NA	NA	207 (34.2)	54 (8.9)	10 (45.4)	3 (13.6)
Yes	NA	NA	11 (36.7)	7 (23.3)	2 (40.0)	NA
Age at first sex						
≤18	246 (25.6)	95 (9.8)	NA	NA	535 (52.4)	45 (4.4)
≥18	342 (22.8)	111 (7.4)	NA	NA	761 (50.2)	67 (4.4)
Had access to condom inside prison						
No	337 (25.7)	82 (6.2)	332 (21.5)	116 (7.5)	593 (51.7)	49 (4.3)
Yes	114 (25.5)	27 (6.0)	132 (37.8)	14 (4.0)	301 (57.6)	14 (2.7)
HIV knowledge ^2^						
Insufficient	520 (21.8)	180 (7.6)	418 (18.7)	162 (7.3)	950 (46.3)	89 (4.3)
Sufficient	141 (28.3)	43 (8.6)	185 (32.3)	49 (8.5)	492 (60.1)	30 (3.7)
Self-perceived risk of HIV ^3^						
No	317 (23.2)	123 (9.0)	267 (19.6)	106 (7.8)	831 (51.6)	71 (4.4)
Yes	301 (24.1)	86 (6.8)	288 (26.2)	85 (7.7)	535 (48.7)	44 (4.0)

^1^ Temporary marriage

^2^ Sufficient knowledge about the transmission of HIV

^3^ The risk of contracting HIV from the individual's point of view

*Not applicable

**Fig. 1:**
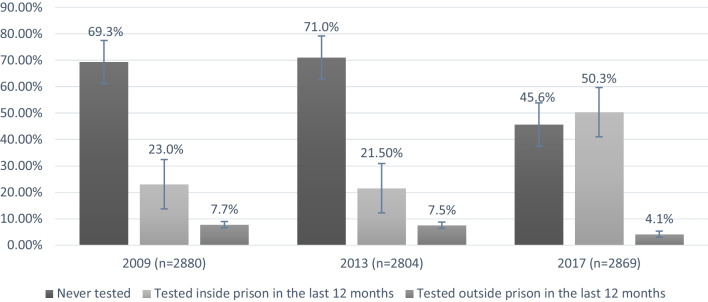
HIV testing among incarcerated people with a history of high-risk behaviours ^a^(P-value < 0. 001). ^a^HIV-related High-risk behaviours were considered a history of injection drug use (ever), having multiple sex partnerships (the last 12 months), or having had a tattoo (ever)

Among 2,880 included participants in 2009, 661 (23.0%, 95% CI: 21.4, 24.5) participants had tested for HIV inside the prison in the last 12 months. Also, 223 (7.7%, 95% CI: 6.8, 8.7) participants had tested for HIV outside the prison in the last 12 months. Of the 2,804 included participants in 2013, 603 (21.5%, 95% CI: 20.0, 23.1) had a history of HIV test inside the prison in the last 12 months, and 211 (7.5%, 95% CI: 6.6, 8.5) had a history of HIV test outside the prison in the last 12 months. In 2017, of the 2,869 included participants, 1,442 participants had a history of HIV test inside the prison in the last 12 months (50.3%, 95% CI: 48.4, 52.1) and 119 (4.1%, 95% CI: 3.5, 4.9) participants with an HIV test outside the prison in last 12 months (Fig. 1).

### Correlates of having an HIV test in the last 12 months inside and outside prison

In the bivariable regression model, sex, age at interview, current marital status, educational level, history of previous incarceration, lifetime history of drug use, current receipt of MMT, having access to condoms inside prison, and sufficient HIV knowledge were significantly associated with having an HIV test in the last 12 months inside prison. In addition, age at the interview, current marital status, educational level, and sufficient HIV knowledge were associated with having an HIV test in the last 12 months outside prison (Table 2).

**Table 2:** Bivariable multinomial logistic regression on HIV testing in the last 12 months among incarcerated people (n = 8553)

Variable	HIV testing inside and outside prison in the last 12 months			
	Inside prison vs. never tested		Outside prison vs. never tested	
	RRR (95% CI)	P-value	RRR (95% CI)	P-value
Study year				
2009	0.30 (0.26, 0.33)	<0.001	1.22 (0.97, 1.55)	0.084
2013	0.27 (0.24, 0.30)	<0.001	1.16 (0.92, 1.47)	0.202
2017	1		1	
Sex				
Male	0.50 (0.38, 0.66)	<0.001	1.07 (0.55, 2.07)	0.823
Female	1		1	
Age at interview		<0.001		0.046
≤29	1		1	
>29	1.83 (1.65, 2.03)		1.20 (1.00, 1.44)	
Current marital status				
Single	1		1	
Married	0.98 (0.87, 1.01)	0.784	1.03 (0.83, 1.29)	0.731
Divorced, widowed, or sigheh	0.63 (0.54, 0.73)	<0.001	1.29 (1.01, 1.64)	0.039
Educational level				
Illiterate or primary school	1		1	
Secondary or high school	1.38 (1.24, 1.53)	<0.001	1.55 (1.26, 1.91)	0.001
College education	0.75 (0.66, 0.85)	<0.001	1.54 (1.22, 1.94)	0.001
History of previous incarceration				
No	1		1	
Yes	1.58 (1.43, 1.75)	<0.001	1.14 (0.94, 1.37)	0.159
Lifetime history of drug use				
No	1		1	
Yes	2.12 (1.82, 2.46)	<0.001	1.20 (0.93, 1.54)	0.143
Currently receiving MMT inside prison				
No	1		1	
Yes	2.48 (2.20, 2.79)	<0.001	1.15 (0.93, 1.43)	0.178
Had access to condom inside prison				
No	1		1	
Yes	1.50 (1.32, 1.71)	<0.001	0.77 (0.57, 1.05)	0.101
HIV knowledge				
Insufficient	1		1	
Sufficient	1.98 (1.78, 2.20)	<0.001	1.29 (1.04, 1.60)	0.017
Self-perceived risk of HIV				
No	1.01 (0.91, 1.11)	0.806	1.12 (0.93, 1.34)	0.221
Yes	1		1	

Our multivariable regression model showed that > 29 years of age (Relative-risk ratio (RRR): 1.24, 95% CI: 1.05, 1.47), history of previous incarceration (RRR: 1.46, 95% CI: 1.24, 1.71), current receipt of MMT inside prison (RRR: 2.09, 95% CI: 1.81, 2.43), having access to condoms inside prison (RRR: 1.42, 95% CI: 1.20, 1.68), and sufficient HIV knowledge (RRR: 1.74, 95% CI: 1.47, 2.05) were significantly associated with an increased probability of having an HIV test in the last 12 months inside prison. No covariate was significantly associated with the probability of having an HIV test in the last 12 months outside prison (Table 3).

**Table 3:** Multivariable Multinomial logistic regression on HIV testing in the last 12 months among incarcerated (n = 8529)

Variable	HIV testing inside and outside prison in the last 12 months			
	Inside prison vs. never tested		Outside prison vs. never tested	
	RRR (95% CI)	P-value	RRR (95% CI)	P-value
Age at interview				
≤29	1		1	
>29	1.24 (1.05, 1.47)	0.010	1.14 (0.82, 1.58)	0.418
History of previous incarceration				
No	1		1	0.289
Yes	1.46 (1.24, 1.71)	< 0.001	1.18 (0.86, 1.63)	
Currently receiving MMT inside prison				
No	1		1	0.102
Yes	2.09 (1.81, 2.43)	< 0.001	1.27 (0.95, 1.70)	
Had access to condom inside prison				
No	1		1	
Yes	1.42 (1.20, 1.68)	< 0.001	0.74 (0.50, 1.09)	0.137
HIV knowledge				
Insufficient	1		1	
Sufficient	1.74 (1.47, 2.05)	< 0.001	1.11 (0.78, 1.59)	0.528

## Discussion

We assessed HIV testing among incarcerated people with a history of HIV-related high-risk behaviours in Iran. We found that in 2009 and 2013, about one-fifth of the eligible participants had a history of HIV testing inside the prison; however, half of the eligible incarcerated people had a history of HIV testing inside the prison in 2017. More incarcerated people in this study had tested inside rather than outside prisons. While this could be partly due to the improvement of harm reduction programs inside prison settings and the availability of HIV testing in Iran’s prisons [15], it could also point to potential structural barriers to testing and socio-economic vulnerabilities among incarcerated people before or after incarceration. Indeed, there was no significant association between HIV testing outside the prison and related variables. HIV testing outside the prison was lower, with around 7% prevalence in 2009 and 2013 and 4% in 2017. We found incarcerated people who were older, had a history of previous incarceration, were currently receiving MMT inside prison, had access to condoms inside prison and sufficient knowledge about HIV transmission, and had a higher chance for HIV testing inside the prison. Also, there was no significant association between HIV testing outside the prison and the main variables.

We found that HIV testing inside prisons had increased in 2017 compared to the 2009 and 2013 surveys. This increase in HIV testing could be due to improved prison harm reduction programs. Indeed, triangular clinics inside prisons in Iran provide HIV testing and counseling and educational materials about HIV prevention. Such facilities were vital in reaching high-risk incarcerated people and controlling the HIV epidemic in the early 2000s inside prisons in Iran [15]. Although the impact of expanding harm reduction services inside prisons has not yet been systematically evaluated, the result of a modelling study in Iran suggests the interventions inside prisons may have reduced the national HIV transmission rate and highlights the importance of continued investments in these services and interventions inside prisons [30]. Given the existing gap in HIV testing uptake, following an opt-out testing approach could be beneficial inside prisons in Iran.

We also found that HIV testing inside the prisons was higher among older people. Age could influence HIV testing via different pathways (e.g., through increased awareness of HIV risks) [31, 32], and lower levels of HIV testing among younger men have reflected their lower perception of risk [33]. However, the impact of age on HIV testing is equivocal in the literature. For example, a study in 2003 in Ontario, Canada, suggested that getting older was negatively associated with HIV testing among incarcerated people [34]. However, Rosen et al. showed that HIV testing increased with age among incarcerated people in North Carolina [35]. Our findings highlight the need for age-specific interventions inside prisons to ensure younger people are well-informed about HIV-related risk behaviours and the importance of routine HIV testing.

We also found that incarcerated people with a history of previous incarceration, currently receiving MMT inside prison, access to condoms inside prison, and sufficient knowledge about HIV transmission had a higher probability of HIV testing inside the prison. This finding highlights that harm reduction services inside prisons might have been impactful in encouraging incarcerated people with a higher risk of drug use and sexual behaviour profiles to engage in HIV testing; as shown by our findings in 2009, the prevalence of HIV testing among the incarcerated people has remained low [36]. However, the association between sufficient knowledge about HIV transmission and HIV testing is interesting and could be a helpful finding to control HIV in the context of this population in Iran. Indeed, those with sufficient knowledge about HIV transmission may prefer to test for HIV, and HIV testing could itself increase their HIV knowledge. The findings of a systematic review indicated that being tested without wanting an HIV test was associated with lower HIV knowledge [37]. Moreover, elevating HIV knowledge creates motivation for risk reduction and has been associated with HIV testing and treatment uptake [38]. Therefore, incarcerated people must be supported and encouraged to take advantage of education, services, and facilities for reducing HIV infection and transmission to others.

We acknowledge the limitations of our study. First, the cross-sectional nature of our surveys limited the drawing of any causal inference in the observed associations. Second, the self-reported nature of our data makes it prone to social desirability and recall biases. As some of the behaviours asked in the interview were sensitive (e.g., homosexual sex, sex work, or injection behaviors), some people may not have disclosed their behaviors. Third, we did not measure the availability of antiretroviral treatment after HIV testing for people living with HIV, which could be explored in future studies. Lastly, we did not measure the barriers to HIV testing inside and outside the prisons. We recommend future quantitative and qualitative studies to examine the obstacles to HIV testing among incarcerated people.

## Conclusion

While the increasing prevalence of HIV testing inside prisons in Iran is encouraging, our findings indicate that incarcerated people’s HIV testing practices are still considerably low both inside and outside prisons in Iran. Our findings highlight the need to revisit and re-evaluate existing HIV testing policies and services inside and outside prison settings to help improve HIV testing uptake among this subpopulation. Scaling up rapid tests and routine opt-out HIV testing services could help encourage incarcerated people to further use the available harm reduction facilities inside and outside prisons.

## Supplementary Information

**Additional file 1.** Multivariable Multinomial logistic regression on HIV testing in thelast 12 months among incarcerated in 2009, 2013, and 2017.

## Data Availability

The Ministry of Health and Medical Education (MOH) of Iran and the Iranian prison office own the data. After the permission of the MOH and Prison office, data will be available upon request submitted to info@hivhub.ir (the HIV/STI Surveillance Research Center).

## Abbreviations

RRR: Relative-risk ratio
PWID: People who inject drugs
MMT: Methadone maintenance therapy
FSW: Female sex workers
BBSS: Bio-behavioural surveillance surveys
CI: Confidence Intervals
TCs: Triangular clinics
