# Supplementary material for: HIV testing among incarcerated people with a history of HIV-related high-risk behaviours in Iran: Findings from three consecutive national bio-behavioural surveys
Source: BMC Infect Dis. 2022 Dec 5;22:907. doi: 10.1186/s12879-022-07897-z (PMC9721074; doi:10.1186/s12879-022-07897-z)
Supplement: Supplementary file 1 — Additional file 1. Multivariable Multinomial logistic regression on HIV testing in thelast 12 months among incarcerated in 2009, 2013, and 2017. [file 12879_2022_7897_MOESM1_ESM.docx]

**Additional file 1**

**Multivariable Multinomial logistic regression on HIV testing in the last 12 months among incarcerated in 2009**

| **Variable** | **HIV testing inside and outside prison in the last 12 months** | | | |
| --- | --- | --- | --- | --- |
|  | **Inside prison vs. never tested** | | **Outside prison vs. never tested** | |
|  | **RRR (95% CI)** | **P-value** | **RRR (95% CI)** | **P-value** |
| **Age at interview** |  |  |  |  |
| ≤29 | 1 |  | 1 |  |
| >29 | 1.27 (1.01, 1.61) | 0.040 | 1.38 (0.95, 2.00) | 0.082 |
| **Currently receiving MMT inside prison** |  |  |  |  |
| No | 1 |  | 1 | 0.463 |
| Yes | 2.37 (1.86, 3.02) | <0.001 | 1.14 (0.79, 1.63) |  |
| **HIV knowledge** |  |  |  |  |
| Insufficient | 1 |  | 1 |  |
| Sufficient | 1.41 (1.07, 1.86) | 0.014 | 0.96 (0.60, 1.53) | 0.879 |
|  |  |  |  |  |

**Multivariable Multinomial logistic regression on HIV testing in the last 12 months among incarcerated in 2013.**

| **Variable** | **HIV testing inside and outside prison in the last 12 months** | | | |
| --- | --- | --- | --- | --- |
|  | **Inside prison vs. never tested** | | **Outside prison vs. never tested** | |
|  | **RRR (95% CI)** | **P-value** | **RRR (95% CI)** | **P-value** |
| **History of previous incarceration** |  |  |  |  |
| No | 1 |  | 1 | 0.647 |
| Yes | 1.61 (1.21, 2.13) | 0.001 | 0.90 (0.57, 1.10) |  |
| **Currently receiving MMT inside prison** |  |  |  |  |
| No | 1 |  | 1 | 0.149 |
| Yes | 2.10 (1.66, 2.65) | <0.001 | 1.36 (0.89, 2.08) |  |
| **Had access to condom inside prison** |  |  |  |  |
| No | 1 |  | 1 |  |
| Yes | 2.01 (1.54, 2.63) | <0.001 | 0.66 (0.35, 1.23) | 0.193 |
| **HIV knowledge** |  |  |  |  |
| Insufficient | 1 |  | 1 |  |
| Sufficient | 2.02 (1.55, 2.63) | <0.001 | 1.35 (0.82, 2.21) | 0.224 |
|  |  |  |  |  |

**Multivariable Multinomial logistic regression on HIV testing in the last 12 months among incarcerated in 2017.**

| **Variable** | **HIV testing inside and outside prison in the last 12 months** | | | |
| --- | --- | --- | --- | --- |
|  | **Inside prison vs. never tested** | | **Outside prison vs. never tested** | |
|  | **RRR (95% CI)** | **P-value** | **RRR (95% CI)** | **P-value** |
| **Age at interview** |  |  |  |  |
| ≤29 | 1 |  | 1 |  |
| >29 | 1.33 (1.05, 1.69) | 0.015 | 0.97 (0.54, 1.76) | 0.939 |
| **History of previous incarceration** |  |  |  |  |
| No | 1 |  | 1 | 0.094 |
| Yes | 1.53 (1.23, 1.91) | <0.001 | 1.68 (0.91, 3.09) |  |
| **Had access to condom inside prison** |  |  |  |  |
| No | 1 |  | 1 |  |
| Yes | 1.26 (1.01, 1.57) | 0.036 | 0.75 (0.40, 1.41) | 0.386 |
|  |  |  |  |  |
